# Supplementary figures and images for: Salivary MicroRNA Signature for Diagnosis of Endometriosis
Source: J Clin Med. 2022 Jan 26;11(3):612. doi: 10.3390/jcm11030612 (PMC8836532; doi:10.3390/jcm11030612)

## Annex S2 : MIRNome Sequencing Analysis Pipeline adaptation from Potla et al. (45)

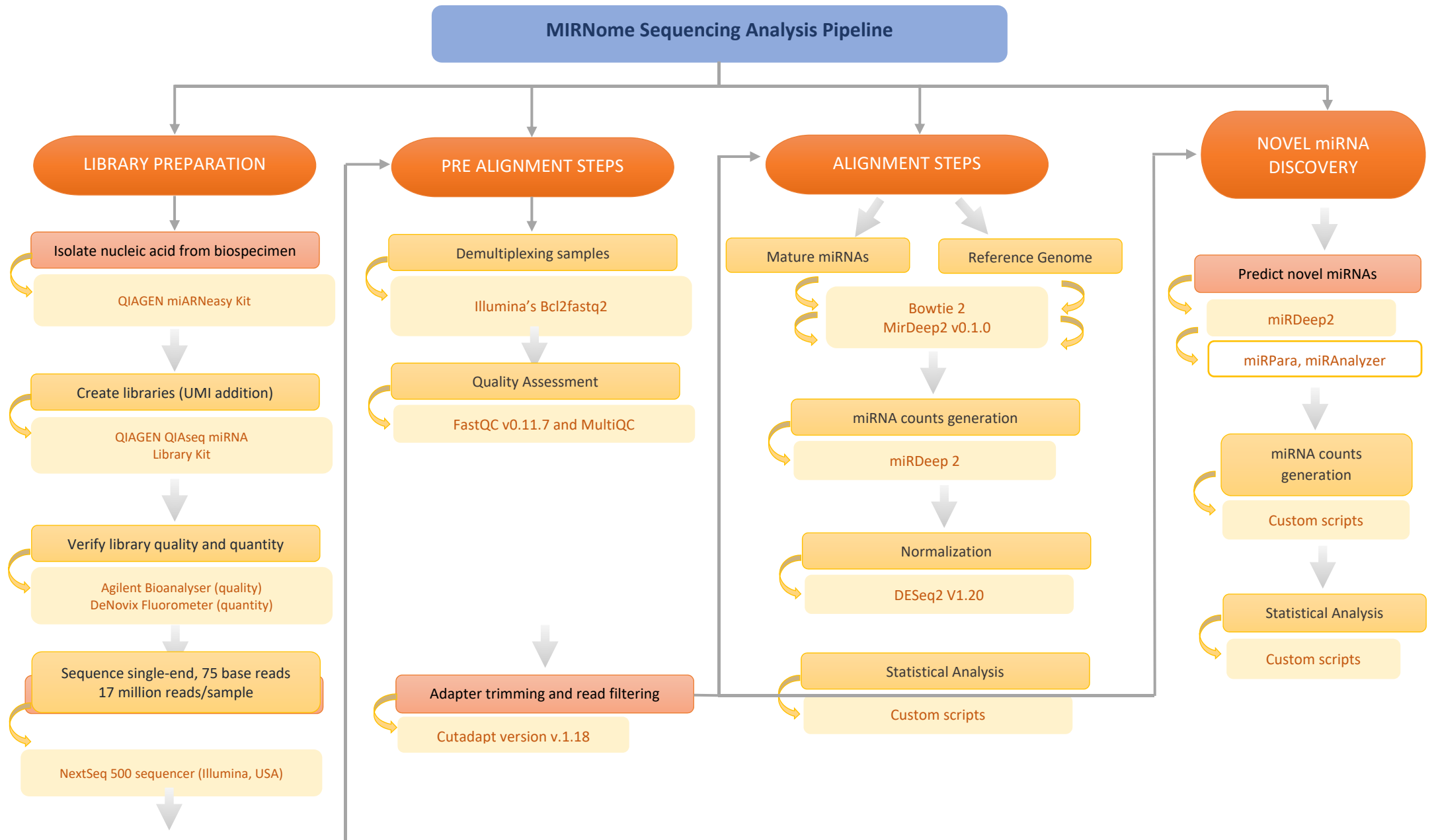

Supplement: Supplementary file 1 [file jcm-11-00612-s001.zip › Annex S2 salive.pdf]
